# Evaluation of a Virtual Dance Class for Cancer Patients and Their Partners during the Corona Pandemic—A Real-World Observational Study

**DOI:** 10.3390/curroncol30050337

**Published:** 2023-04-24

**Authors:** Jutta Hübner, Ivonne Rudolph, Tobias Wozniak, Ronny Pietsch, Mascha Margolina, Isabel Garcia, Katharina Mayr-Welschlau, Thorsten Schmidt, Christian Keinki

**Affiliations:** 1 Medizinische Klinik II, Hämatologie und Internistische Onkologie, Universitätsklinikum Jena, Am Klinikum 1, 07747 Jena, Germany; 2 Waldburg-Zeil Kliniken, Rehabilitationsklinik Bad Salzelmen, Badepark 5, 39218 Schönebeck, Germany; 3 Working Group Prevention and Integrative Oncology, German Cancer Society, Kuno-Fischer-Str. 8, 14057 Berlin, Germany; 4 Cancer Center North, University Hospital Schleswig-Holstein, Campus Kiel, Arnold-Heller-Straße 3, 24105 Kiel, Germany

**Keywords:** neoplasm, supportive care, physical activity, dancing, quality of life, virtual training

## Abstract

Background: During the corona pandemic, all courses on physical activity for cancer patients were canceled. The aim of our study was to evaluate the feasibility of switching dancing classes for patients and their partners to online classes. Methods: Patients and partners from courses at four different locations who consented to the online course offer were asked to fill in a pseudonymous questionnaire on access to the training, technical challenges, acceptance and well-being (1-item visual analog scale from 1 to 10) before and after the training. Results: Sixty-five participants returned the questionnaire (39 patients and 23 partners). Fifty-eight (89.2%) had danced before, and forty-eight (73.8%) had visited at least one course of ballroom dancing for cancer patients before. The first access to the online platform was difficult for 39 participants (60%). Most participants (57; 87.7%) enjoyed the online classes, but 53 (81.5%) rated them as less fun than the real classes as direct contact was missing. Well-being increased significantly after the lesson and remained improved for several days. Conclusion: Transforming a dancing class is feasible for participants with digital experience and goes along with technical difficulties. It is a substitute for real classes if mandatory and improves well-being.

## 1. Introduction

Physical activity is one of the most important supportive methods for cancer patients. Being physically active helps to cope with diseases, reduces the side effects of treatments and improves the quality of life [1,2,3]. Studies showed significant improvements in various physical and psychological symptoms, e.g., fatigue, pain, dyspnea, and sleep [1]. In addition, physical activity can improve the immune system [2]. Moreover, patients who are physically active also have a better prognosis [4]. In their systematic review, Friedenreich et al. were able to calculate that the risk of mortality can be reduced through physical activity (hazard ratio = 0.63, 95% confidence interval = 0.53–0.75) [4]. 

As a consequence, guidelines recommend regular training for all patients and survivors [5,6]. Furthermore, it was found that physical activity has a positive effect on the relationship between patients and relatives. Physical activity leads to a positive improvement in confidence in the fight against cancer by relatives [3]. This can strengthen the emotional relationship between patients and relatives.

However, there are several barriers that reduce adherence to below the level recommended in most patients. Barriers are a lack of information [7] but also pain and other side effects in case of insufficient supportive care or false training supervision. Romero et al. reported the most common barriers as fatigue (78%), pain (71%), difficulty becoming motivated (67%) or remaining disciplined (65%) [8].

In 2016, we started a ballroom dancing project for cancer patients as this activity is often not regarded as a sport but a social activity. It allows integration of the partner and offers the possibility to tailor the intensity of the movements so that participants in one course may exercise with more or less intensity. This allows patients to take part in a lesson even on days they feel less powerful or even fatigued [9,10,11].

Dance movement therapy has been shown to lead to physical, mental and social benefits and has been evaluated mostly as individual dancing [12,13,14]. As elaborate assessments [15,16] and a high number of hours of training per week reduce adherence, our concept is a low-level, highly motivating course once per week continuously over the whole year [9,10,11]. The frequency and duration of the lessons were adopted from the former real classes. The national guideline for complementary and alternative medicine for oncological patients recommends 150 min of moderate (or 75 min of vigorous) physical activity per week and a mixture of endurance and strength training [4], half of this time as dancing was found appropriate to allow participants to choose a supplementing training.

As a broad range of dancing (Standard and Latin-American) and music styles are included, most people find styles they enjoy. Moreover, they are able to attend classes at any regular dancing school and open classes or events so that they are not confined to the patient course. 

Evaluations have shown the feasibility of this training and provided the first data on improving well-being and fatigue [17]. With workshops in other cities, new groups were started. In 2020, the corona pandemic forced us to close the groups. 

During the Corona lockdowns, healthy people also significantly reduced their physical activity. This was also the case in cancer patients. In a large study, a virtual exercise program with patients during antineoplastic treatment increased not only feasibility but also endurance, quality of life and feelings of support, and decreases in loneliness and fatigue were shown [5].

While courses for gymnastics, yoga, etc., were first offered online, ballroom dancing seemed not feasible mostly for reasons of space needed. However, with Germany being in a further lockdown in January 2021, a decision to start online lessons was made. The objective of our accompanying study was to evaluate the feasibility of a digital course on ballroom dancing. With a positive evaluation, a continuous digital course would provide access for patients living in regions without a similar offer also after the pandemic.

## 2. Materials and Methods

### 2.1. Patients

All patients and their partners from the former courses in four cities were invited to online lessons. We included adult patients with a cancer diagnosis and their partners (which could be a spouse, another member of the family or a household). Inclusion criteria were any type of malignancy; patients during or after cancer treatment; an interest in the course; the ability to perform exercises; a means of access to the digital platform; a loudspeaker; a screen; and, if possible, a camera and about one square meter of place to move. Exclusion criteria were inability to understand the German instructions or not being able or willing to fill in the questionnaire. Patients could also attend the lessons without a partner. With one experienced trainer, we opened a class for newcomers. We included all patients who started training in the first courses. Recruitment took place from January to March 2021.

### 2.2. Training

The training consisted of 60 to 70 min of training and a short introduction and final round of welcome and goodbye. The course consisted of 12 lessons, one each week. Movements came from Standard and Latin American dances and were adapted to little room available and dancing alone. Most movements were taught as single dancing, but experienced couples could transfer them to steps for a couple in case they had enough space. The trainers were experienced trainers for ballroom dancing, and all had worked with cancer patients in real classes before and had attained a certificate from the Working Group Prevention and Integrative Oncology of the German Cancer Society. The detailed plan of every lesson was determined by the trainer and was highly dependent on the prior knowledge and skills of the participants and the individual and group progress during the course.

The online courses took place on a safe browser-based system that did not need a login password but only the link, which was the same for all lessons during the course.

### 2.3. Questionnaire

We developed a standardized questionnaire consisting of the following:

- A short demographic part;
- A section with questions on experience with the Internet and technical access;
- Questions on the effort needed to access the digital platform;
- Feasibility of the dancing at home (space, noise and neighbors);
- Satisfaction with the course and comparison to the real classes;
- Well-being before, during and after the lessons in the first and last week of a 12-week course.

The first draft of the questionnaire was set up by JH (integrative oncology) after discussing the project of digitalization of the dance classes and the accompanying research question with three experts (IR (rehabilitation medicine), TS (sports medicine), CK (physician) and the trainers (TW, RP, MM, IG and KMW). All experts and trainers revised the first draft, and a consensus was made in a digital meeting.

Most questions were closed questions offering several answers or questions to agree or disagree on a Likert scale from 1 (I very much agree) to 4 (I do not agree at all).

For well-being, we used the same 1-item scale with a visual analog scale from 1 (very well) to 10 (not well at all), which we had used before in the normal courses [11].

### 2.4. Statistics

We utilized IBM SPSS Statistics 25 for the data collection and statistical analysis. For associations, we used the Chi-Square test, and to analyze the influence of dancing on well-being, we used the asymptotic Wilcoxon test. In both, *p* < 0.05 was considered significant. To determine effect sizes for well-being, we calculated Pearson’s correlation coefficient r.

### 2.5. Ethics Vote

The study was approved by an ethics committee of a university hospital in Germany.

## 3. Results

### 3.1. Demographic Data

All in all, 65 patients and partners from 4 cities in Germany took part in the study (see Table 1). Thirty-nine (60.0%) were patients, and twenty-three (35.4%) were partners. Ten patients were male (15.4% of all participants), and 29 were female (44.6%), while 15 partners were male (23.1%), and 8 partners were female (12.3%). Most patients had breast cancer (*n* = 17; 43.6% of patients).

Forty-three (66.1%) participated in the lessons with their spouse, four (6.2%) with a member of the family and three (4.6%) with a friend. All in all, 13 (20.0%) were singles. Only 7 participants (10.8%) had never attended a dancing lesson before, while 58 (89.2%) had some experience. Concerning the former real classes for cancer patients, 48 (73.8%) had attended at least one course, while 15 (23.1%) marked being newcomers. 

### 3.2. Access to Virtual Dance Lessons 

Most of the participants had experiences with the Internet, and 50 (76.9%) marked that they were online daily. Eleven (16.9%) used the Internet several times a week, and only four (6.1%) used it several times a month or even less often. Most participants (60; 75.4%) reported that their Internet was fast enough, while only four (6.2%) thought it to be too slow. For normal access to the Internet, most (57; 87.1%) used a PC, and 50 (76.9%) used a tablet (see Table 2). For the classes, there was a clear preference for the PC or laptop and not for tablet or smartphone. 

### 3.3. Experiences and Satisfaction with the Online Lessons 

**Figure 1:**
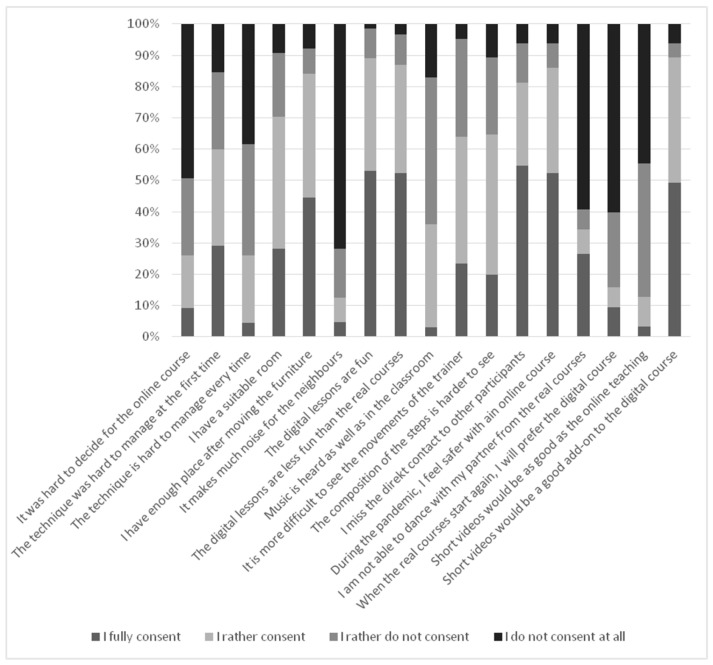
Experiences and satisfaction with the online lessons (*n* = 65).

For most participants, the decision to take part in the courses during the pandemic was easy (see Figure 1). Only 6 (9.2%) fully stated that it was difficult, and 11 (16.9%) partially agreed. During the first time, 39 participants (60.0%) found it difficult to access the browser-based system; however, during the course, only a quarter (17 participants, 26.1%) found it difficult every time again. 

Older participants (>55 years) more often declared difficulties with first access to the platform (*p =* 0.039). However, after the first access, they did not report more problems in the consequent lessons than younger participants. While they enjoyed the lessons as much as the younger ones, they more often reported having less fun than in the real classes (*p =* 0.001). Accordingly, they more often missed direct contact with other participants (*p =* 0.009). However, they also more often thought that the online course was safer during the pandemic (*p =* 0.10). Patients and healthy partners did not rate safety differently.

While 57 (87.7%) stated that the lessons were fun, nearly the same stated that it was less fun than the real courses (*n* = 53; 81.5%). The quality of the sound of the music was acceptable, but most participants reported that watching the movements of the trainers and understanding the steps was rather difficult (“movements hard to see”: 15 fully agreed (23.15), 26 partially agreed (40.4%); “steps hard to understand”: 13 fully agreed (20.0%), 29 partially agreed (44.6%)). While most felt safer in a digital class during the pandemic, most also missed direct contact with other patients (52; 79.0%), and only eight (12.3%) thought that they would consider digital courses after the pandemic. 

### 3.4. Well-Being before and after the Training 

**Figure 2:**
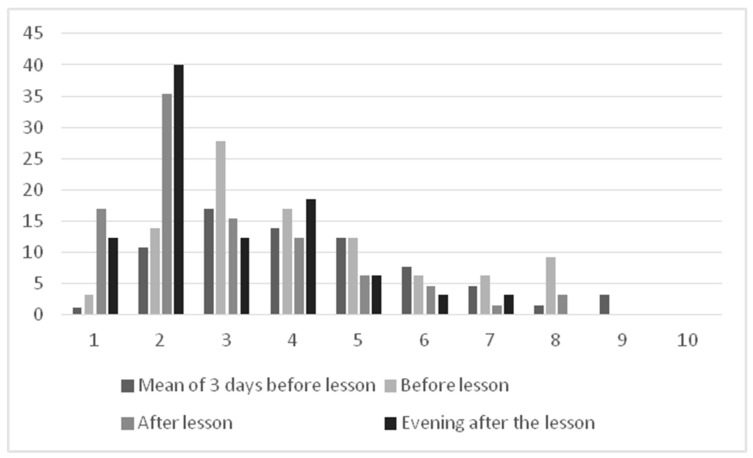
Well-being of the participants (self-rating on a visual analog scale from 1 = very well to 10 not well at all; *n* = 61).

Overall, well-being was rather heterogeneous in this group of patients and partners. When comparing well-being before and after the training, there was a significant improvement from the last 3 days to after the course, which lasts for 5 days (immediately after the lesson: z = −5.190, *p* < 0.001, r = 0.66; in the evening: z = −5.286, *p* < 0.001, r = 0.68; in the evening of the first day after the lesson: z = −5.307, *p* < 0.001, r = 0.68; in the evening of the third day after the lesson: z = −2.897, *p =* 0.004, r = 0.37; in the evening of the fourth day after the lesson: z= −2.551, *p =* 0.011, r = 0.33. Well-being returned to the initial values only on day 5 (see Figure 2).

**Figure 3:**
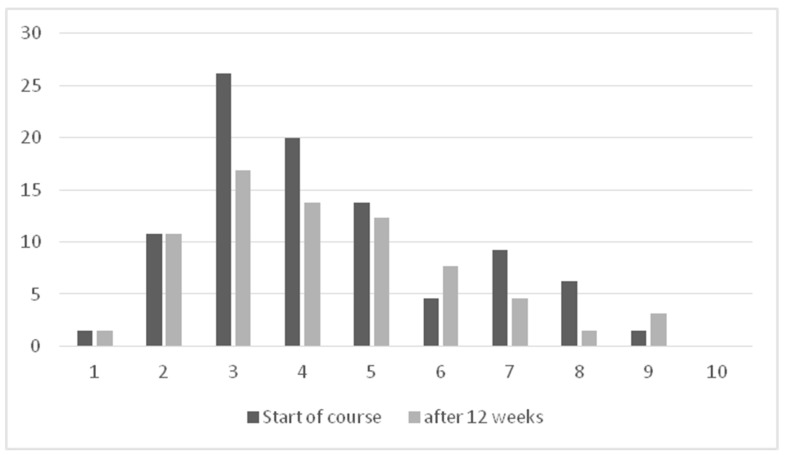
Well-being before start of course and after 12 weeks (self-rating on a visual analog scale from 1 = very well to 10 not well at all; mean of 3 days before lesson; *n* = 61).

At the end of the course in week 12, comparing well-being assessed on the three days before the last lesson and after the lesson, there was a significant improvement, which remained throughout the whole week (Figure 3): immediately after the lesson: z = −4.204, *p* < 0.001, r=0.54; in the evening: z = −3.717, *p* < 0.001, r = 0.48; in the evening of the first day after the lesson: z = −3.678, *p* < 0.001, r = 0.47; and in the evening of the sixth day after the lesson: z = −2.565, *p* < 0.010, r= 0.33. Neither age, gender, status (patient versus partner) nor current cancer treatment had an influence on this improvement.

## 4. Discussion

In our study, 65 participants took part in a 12-week online dance course. We had more patients than healthy partners in the courses, in part because, in some couples, both partners were survivors of cancer. Moreover, several patients took part alone. Nearly half of the patients had participated in former real classes, but newcomers could also be integrated into the online lessons.

Participating in digital dancing lessons with a focus on ballroom style significantly improved well-being, starting immediately after the training and remaining through the whole week. As neither age, gender, status (patient versus partner) nor current cancer treatment had an influence on this improvement, the training seems to be adequate for a large variety of patients and partners. The advantages of a closed lesson for patients and relatives were shown to be important in our first study as patients take more time to learn the steps and benefit from the individual adaptability of the intensity of movements [6]. The chosen duration of 60 to 75 min is 15 to 30 min below that of our real courses and seems adequate for the format. Therefore, the duration of the digital course is less than that recommended in the guideline [4]. For this reason, the digital offer is to be seen as a component of an otherwise active lifestyle. Concerning well-being, the result is stronger than in our pilot study of a real course [7]. One explanation might be that for the participants did not have other supervised training options several months. It may also be due to the digital format, which inevitably means that patients have to train at their own homes. Well-being is usually better at home than in a foreign training place. 

About a quarter of the participants reported finding the decision to take part in an online course difficult, as barriers are different from barriers to real classes. For the latter, participants have to invest time and money to come to the location. An online course may be easier to access for those who are familiar with the techniques and have suitable access to the Internet. Moreover, people with a higher socioeconomic background may find it easier to participate as they are more likely to have a home with enough space and comfortable devices with a larger screen. The barrier to accessing the digital platform was rather high at the start, but most participants were able to access the system on their own later on. However, for about a quarter, access was difficult to manage for each lesson. Since it is not known how many persons were interested but did not manage to join the class and/or contact us, we were not able to rate the relevance of all barriers for all cancer patients. To better integrate people with lower digital competencies, a time slot of several minutes before the start and a helpdesk might be valuable as, for the trainers, it is difficult to manage technology as well as training support. 

Overall, our study shows that while the participants accepted the digital format, the great majority prefer real classes and contact with other participants, not only via chat or microphone. In fact, for motivating people to perform physical activity, the feeling of a community is highly important [18]. This group feeling is especially very important for cancer patients, even more so for cancer patients exercising at home in digital lessons [19,20,21]. Firstly, it helps not to feel lonely. Secondly, it gives hope that one might succeed with the exercises as others do. Thirdly, in a group of cancer patients, everybody understands the floating levels of energy and mood. In contrast, in mixed groups or groups of healthy people, cancer patients often have to explain why they have to pause or why they look depressed, which reminds them of their disease and the debilitating sequels.

With respect to the reliability of the data, there are several concerns: first, the group of participants is not representative, as most probably all participants were interested in dancing. However, it would not make sense to include patients without any interest in a study on a special type of physical activity at all. Second, well-being was rated using a single-item VAS, which is a rather rough instrument but keeps the drop-out rate rather low. One might ask whether former experiences with dancing were important, but for the patients without former participation, the results were quite similar. Moreover, all patients had paused for at least six months due to the lockdown during the pandemic. In fact, the effect size was even higher and longer-lasting at the end of the course after 12 weeks. Another explanation might be the social desirability of the answer, which might have let patients mark better well-being after the lessons. However, the long duration and even better results at the end of the course speak against this notion. From a statistical point of view, it must also be considered that skills in using the Internet may have had an influence because the patients in the baseline survey differed in this regard. Even if an influence could possibly exist, no additional skills in dealing with the Internet are required after the start of the digital courses. After starting the digital courses, the focus is on physical activity, which is why we think an influence is conceivable but not very likely. Subgroup analyses could be carried out in a larger collective. This study was mainly concerned with feasibility, which is why additional statistical studies were not carried out. One question remains open: is it a specific effect, or is it the effect of social activity? In fact, dancing is more than physical activity. It combines partnership, social contact in the group, and physical and cognitive activity. In this combination, dancing is a quite unique activity that fulfills the premises of a combined body–soul–mind intervention. 

There are two more limitations to our study. Firstly, the questionnaire was not validated, nor was its reliability assessed before starting the study. This was due to the sudden development of the pandemic and the urgent need to offer training for the patients. However, the lack of validity testing of the questionnaire used is a major limitation of the findings. In future research, there is a need to assess the validation of the used questionnaire before the findings can be generalized.

Second, the sample is rather small, yet larger than other research focusing on dancing with cancer patients. Moreover, as there were four different trainers, there were most probably differences between the classes, which will be analyzed in a second article. However, a sensitivity analysis did not reveal any differences between the four different groups.

## 5. Conclusions

In conclusion, our data confirmed the feasibility of digital dancing and especially ballroom dancing classes for cancer patients. Dancing is suitable for patients during treatment and for survivors and even has positive effects on the partners. While most participants prefer real classes and contact with other participants, in cases such as the pandemic, digital offers are a valuable substitute. As barriers to participating in real and virtual classes may differ, a parallel system of both offers might be ideal for reaching patients with different needs and in different conditions. Moreover, with an online version, patients and partners living at places where no dancing school exists may also take place. In fact, such a system would be able to reduce perceived negative aspects of exercise such as cost, transportation, weather, inconvenience, lack of flexibility of courses and safety [22].

The effect sizes we report from our data are strong. For a further study, it would be important to have a longer duration and a pre-planned follow-up. It would be important to establish, as the data suggest, that with a longer duration, the effect might stabilize and improve well-being not only for a few days but continuously. Nevertheless, dancing should be considered a valuable type of physical activity for cancer patients.

## Figures and Tables

**Table 1:** Demographic data (*n* = 65).

		Total	% of All Participants (Patients and Partners)
Age	<30 years	1	1.5
	31–40 years	2	3.1
	41–55 years	15	23.1
	56–65 years	20	30.8
	66–75 years	23	35.4
	>75 years	4	6.2
Gender	Female	38	58.5
	Male	25	38.5
	No answer	2	3.1
Status	Patient	39	60.0
	Partner	23	35.4
	No answer	3	4.6
		Total	% of patients
Cancer	Breast cancer	17	43.6
	Gastrointestinal cancer	6	15.4
	Gynecological cancer	4	10.3
	Others *	9	23.1
Time since diagnosis	<1 month	1	2.6
	1 month–1 year	3	7.7
	1–5 years	23	60.0
	>5 years	11	28.2
	No answer	1	2.6
Current cancer treatment	Yes	18	46.2

* Melanoma, other skin cancer, prostate, thyroid.

**Table 2:** Access to the Internet and the lessons (*n* = 65).

		Number of Participants	%
Normal access to the Internet	PC or laptop	57	87.7
	tablet	50	76.9
	PC at work	19	29.2
	Public Internet	2	3.1
	No regular access	0	0
Access to digital classes via	PC or laptop	56	86.2
	tablet	15	23.1
	PC at work	0	0
	Public Internet	0	0
	No regular access	0	0

## Data Availability

The datasets generated and analyzed during the current study are available from the corresponding author upon reasonable request.
